# Response monitoring of breast cancer patients receiving neoadjuvant chemotherapy using quantitative ultrasound, texture, and molecular features

**DOI:** 10.1371/journal.pone.0189634

**Published:** 2018-01-03

**Authors:** Lakshmanan Sannachi, Mehrdad Gangeh, Hadi Tadayyon, Ali Sadeghi-Naini, Sonal Gandhi, Frances C. Wright, Elzbieta Slodkowska, Belinda Curpen, William Tran, Gregory J. Czarnota

**Affiliations:** 1 Physical Sciences, Sunnybrook Research Institute, Sunnybrook Health Sciences Centre, Toronto, ON, Canada; 2 Department of Radiation Oncology, Odette Cancer Centre, Sunnybrook Health Sciences Centre, Toronto, ON, Canada; 3 Department of Medical Biophysics, Faculty of Medicine, University of Toronto, Toronto, ON, Canada; 4 Division of Medical Oncology, Sunnybrook Health Sciences Centre, Toronto, ON, Canada; 5 Division of General Surgery, Sunnybrook Health Sciences Centre, Toronto, ON, Canada; 6 Department of Anatomic Pathology, Sunnybrook Health Sciences Centre, Toronto, ON, Canada; 7 Division of Breast Imaging, Sunnybrook Health Sciences Centre, Toronto, ON, Canada; Florida State University, UNITED STATES

## Abstract

**Background:**

Pathological response of breast cancer to chemotherapy is a prognostic indicator for long-term disease free and overall survival. Responses of locally advanced breast cancer in the neoadjuvant chemotherapy (NAC) settings are often variable, and the prediction of response is imperfect. The purpose of this study was to detect primary tumor responses early after the start of neoadjuvant chemotherapy using quantitative ultrasound (QUS), textural analysis and molecular features in patients with locally advanced breast cancer.

**Methods:**

The study included ninety six patients treated with neoadjuvant chemotherapy. Breast tumors were scanned with a clinical ultrasound system prior to chemotherapy treatment, during the first, fourth and eighth week of treatment, and prior to surgery. Quantitative ultrasound parameters and scatterer-based features were calculated from ultrasound radio frequency (RF) data within tumor regions of interest. Additionally, texture features were extracted from QUS parametric maps. Prior to therapy, all patients underwent a core needle biopsy and histological subtypes and biomarker ER, PR, and HER2 status were determined. Patients were classified into three treatment response groups based on combination of clinical and pathological analyses: complete responders (CR), partial responders (PR), and non-responders (NR). Response classifications from QUS parameters, receptors status and pathological were compared. Discriminant analysis was performed on extracted parameters using a support vector machine classifier to categorize subjects into CR, PR, and NR groups at all scan times.

**Results:**

Of the 96 patients, the number of CR, PR and NR patients were 21, 52, and 23, respectively. The best prediction of treatment response was achieved with the combination mean QUS values, texture and molecular features with accuracies of 78%, 86% and 83% at weeks 1, 4, and 8, after treatment respectively. Mean QUS parameters or clinical receptors status alone predicted the three response groups with accuracies less than 60% at all scan time points. Recurrence free survival (RFS) of response groups determined based on combined features followed similar trend as determined based on clinical and pathology.

**Conclusions:**

This work demonstrates the potential of using QUS, texture and molecular features for predicting the response of primary breast tumors to chemotherapy early, and guiding the treatment planning of refractory patients.

## Introduction

Neoadjuvant chemotherapy (NAC) is commonly used to treat patients with locally advanced breast cancer (LABC) and a common option for primary operable disease. LABC are large breast tumors greater than 5 cm, including stage 3/4 disease, and in some cases, involve the skin and chest wall. They also typically involve axillary or peripheral lymph nodes and are often surgically unrespectable. The advantage of NAC is that it reduces tumor volume [1] making patients operable and also treats micro-metastatic disease up-front. Unfortunately, not all patients respond well to NAC. Pathological response is a prognostic indicator for long-term disease free survival (DFS) and overall survival (OS); with pathological complete responding patients having better overall survival compared to partially responding patients [2]. This highlights the importance of early detections of ultimate patient responses to cancer therapies. Several studies have indicated that patients who do not respond to initial chemotherapy may benefit from salvage therapies (additional systemic chemotherapy or preoperative radiation or surgery) [3–5], in the case where there is insufficient intra-treatment response. Specifically, in a response-guided neoadjuvant chemotherapy for breast cancer study [4], breast cancer patients were treated with two cycles of docetaxel, doxourubicin, and cyclophophamide (TAC) and randomly assigned early non-responders to four cycles of TAC or vinorelbine and capectiabine (NX) before surgery. In that study, researcher reported that disease free survival was longer in early non-responders receiving TAC-NX than in those receiving TAC x 6. In the Aberdeen study [3], LABC patients underwent CVAP x 4 (cyclophosphamide / vincristine / doxorubicin / prednisone) chemotherapy treatment and randomly assigned early complete and partial responders to further CVAP x 4 or docetaxel x 4. In that study, researcher reported that patients receiving docetaxel had significant higher clinical response rate (94%) than patient receiving CVAP (64%). However, at present, primary tumor response to NAC is evaluated based on changes in tumor size, and histological examinations of the sectioned operative specimen which take place typically months after chemotherapy treatment. This late assessment of treatment response is mainly due to limitations from current imaging modalities, such as magnetic resonance imaging (MRI), x-ray mammography, and conventional ultrasound which mainly rely on morphological-based size changes of tumor. An imaging modality which can assess significant changes in cell death related tumor micro-structure would be advantageous for the early assessment of treatment response.

A significant number of studies have demonstrated that quantitative ultrasound (QUS) techniques may be used to characterize various tissue types, classify tissue abnormalities compared to normal tissues, and differentiate tumor types [6–8]. In preclinical studies, QUS techniques have been demonstrated in the detection tumor response to cancer therapies such as chemotherapy, photodynamic therapy, X-ray radiation therapy and ultrasonically-stimulated anti-vascular microbubble treatment, or combinatorial treatments [9–13]. In a pilot clinical study with a limited number of patients, QUS techniques were used to differentiate clinical/pathological responders and non-responders in an LABC population treated with NAC early after the start of treatment (sensitivity: 77% and specificity: 86% at week 1; sensitivity: 83% and specificity: 100% at week 4) [14,15]. In those studies, QUS parameters such as mid-band fit (MBF), spectral slope (SS) and 0-MHz intercept (SI), average scattering diameter (ASD), and average acoustic concentration (AAC) were investigated and exhibited a strong correlation with primary tumor response. Such parameters reflect tissue micro-structural properties such as scatter size, shape, organization in addition to elastic properties. A recent study has reported an improvement in accuracy in differentiating responder and non-responder groups early at week 1 after NAC initiation (sensitivity: 100% and specificity: 100%) [16] by combining QUS parameter and texture features such as contrast, correlation, energy and homogeneity. These texture parameters quantify the spatial relationship between neighboring regions with respect to acoustic properties. Scatterer spacing (SAS) which is spacing between two adjacent scatterers has also been widely used in tissue characterization applications when tissue contains a detectable periodicity in its structural organization. Recently, it has been used in breast tumor studies such as differentiating normal breast tissue, fibroadenomas, simple carcinomas [17], and characterizing locally advanced breast tumors based on histological grading [18]. Several investigators have evaluated the relationship between breast cancer molecular features and pathological complete response after NAC. In an early stage breast cancer study, researchers investigated the relationship between molecular features and recurrence free survival (RFS) and found a strong correlation of ER, PR, and HER2 receptor scores with RFS (*p =* 0.007–0.019) [19]. Similarly, in an LABC study, investigators have been reported that HER2+ and triple negative breast cancer exhibit higher rate of pathological complete response [2,20]. These finding suggests that molecular subtypes are an important prognostic factor in deciding tumor responses to NAC.

Previous studies by our group have mainly focused on responder (CR+PR) and non-responder classification early after NAC [14,16]. Given data [21] indicating that pathological partial responders have significantly lower overall survival (OS) than pathological complete responders that previous work has been expanded here. In the present study, we examined 96 LABC patients who received NAC studied over a period of 5 years. In the study here, patients were divided into three response groups complete, partial, and non-responders based on combined clinical and pathological responses and, then the correlation of quantitative ultrasound, texture features estimated from primary tumor ultrasound data and clinical/pathological response were investigated. Finally, a multi-feature classification model was developed to differentiate complete responders, partial responders and non-responders early after NAC initiation. This work demonstrates a method to predict three type of treatment response based on mean values of QUS parameters, texture, and molecular features, and could potentially be used in the future to help clinicians personalize NAC for locally-advanced breast cancer.

## Methods

### Study design

This locally advanced breast tumor response monitoring study was approved by the Sunnybrook Research Institute research ethics board. Ninety six locally advanced breast cancer patients were enrolled and all signed an informed consent form before participating in this study. Prior to treatment, as part of their clinical care, MRI images of the breast were acquired for each patient to determine initial tumor size and all patients were subjected to a core needle biopsy to confirm a cancer diagnosis, where information regarding histological subtype and hormone receptors status such as estrogen receptor (ER), progesterone receptor (PR), and human epidermal growth factor receptor 2 (HER2) of tumor were recorded. Physical examination was conducted after each cycle of chemotherapy and size and stiffness of tumor was assessed by clinicians. Post-treatment MRI scans of the breast were also acquired immediately before patient surgery to measure residual tumor size. Axillary lymph node statuses were recorded before and after neoadjuvant chemotherapy. Ultrasound data were collected from each patient at 5 specific times during treatment. The first scan was before the start of chemotherapy and next three scans were at weeks 1, 4, and 8 during treatment, and final scan was one week before mastectomy. After mastectomy, surgical specimens were prepared onto a 5˝×7˝ whole-mount pathology slide and digitized using a confocal scanner (TISSUEscope^TM^, Huron Technologies, Waterloo, ON). A board-certified pathologist examined the specimens and reported the results into the patient’s medical chart. Tumor responses were classified into three groups based on changes detected in primary breast tumor to neoadjuvant chemotherapy as reported in previous imaging studies [22–26]. Patients who had no clinical evidence of tumor in the breast and no histological evidence of invasive carcinoma on pathologic examination of the surgical specimen were classified as complete responders (CR). Patients who had at least a 50% decrease in tumor size and significant decrease in tumor cellularity after treatment were classified as partial responders (PR). Patients who had less than 50% decrease in tumor size accompanied by no significant changes in tumor cellularity were classified as non-responders (NR). Data presented here includes that from a pilot cohort of 35 patients [16].

### Quantitative ultrasound parameter estimation

All radio frequency (RF) ultrasound data were collected using a Sonix RP clinical research system (Analogic Medical Corp., Vancouver, Canada) equipped with a linear array transducer (L14-5/60, Analogic Medical Corp., Vancouver, Canada) with a central frequency of 7 MHz and bandwidth of 4–9 MHz. Data were digitally collected with a sampling frequency of 40 MHz. From each breast tumor, 4 to 6 frames were collected with intervals of 1 cm across the breast, with the transducer focus at the centre of the tumor. The sector size for each image frame was 6 cm along the lateral direction and 4–6 cm along the axial direction.

From each ultrasound frame, several parameters such as MBF (mid-band fit), SS (spectral slope), SI (spectral intercept), ACE (attenuation co-efficient estimate), SAS (spacing among scatterers), ASD (average scatterers diameter) and AAC (average acoustic concentration) were determined using quantitative ultrasound methods. In this technique, tumor regions of interest were selected. Each region of interest was divided into window blocks of size 10λ x 10λ with a 94% overlap in axial and lateral directions. The tumor attenuation (ACE) was calculated using a spectral difference method. A reference phantom technique was used to account for clinical system dependences in quantitative ultrasound parameter estimation. The reference phantom (attenuation: 0.576 dB/MHz/cm; speed of sound: 1488 m/s) was made of 5 to 30 μm glass beads embedded in a homogeneous background of microscopic oil droplets in gelatin (Medical Physics Department, University of Wisconsin, USA). The MBF, SS and SI were calculated by linear regression analysis of the normalized backscatter power spectrum over the frequency bandwidth of the transducer [27]. The scattering spacing, SAS, which represents the distance between adjacent scatterers was determined using an autoregressive spectral analysis method by modeling the tumor echo signal as an autoregressive signal [28]. For SAS parameter estimation, the power spectrum was normalized to that of a plexiglas planar reflector. The ASD and AAC parameters were derived from the backscatter coefficient by comparing measured data with a theoretically derived backscatter coefficient using a Spherical Gaussian scatterer model (SGM) [29]. Finally, colour coded parametric maps for each estimated quantitative ultrasound parameters were developed by generating a spatial map of the parameter values computed over all window blocks.

### Texture analysis

In addition to the mean values of quantitative ultrasound parameters which is determined by averaging QUS parametric map values, spatial distributions of QUS parameters in parametric maps were evaluated using a gray-level co-occurrence matrix (GLCM) [30] method, which represents the angular relationship between neighboring pixels as well as their distances in parametric maps. Sixteen symmetric GLCMs were constructed for each parametric map, considering each pixel’s neighbors located at different distances and directions, i.e, at distances of 1 pixel, 2 pixels, 3 pixels and 4 pixels, and at angles of 0°, 45°, 90°, and 135°. From each symmetric GLCM, four texture features such as contrast (CON), correlation (COR), homogeneity (HOM), and energy (ENE) were determined and averaged. Hence, in this study, a total of 28 textural features (four texture features from MBF, SS, SI, SAS, ASD and AAC parametric maps) were computed. A total of 65 features (7 mean of QUS parameters before treatment, 24 texture features before treatment, 7 changes in mean of QUS parameters after treatment, 24 changes in texture features after treatment, and molecular features such as ER, PR, and HER2) were submitted to a support vector machine (SVM) classifier in order to best determine a three type of response classification.

### Tumor response classification analysis

In order to detect tumor response, a multi-feature response classification was performed on three feature sets such as mean QUS values + texture, molecular features, mean QUS values + texture + molecular features using a support vector machine (SVM) with radial basis function (RBF) kernel classifier. The grid-search on *C* and γ which define the kernel function here was performed for the range of 2^8^, 2^9^, 2^10^,…,2^15^ and 2^−18^, 2^−17^, 2^−16^,…2^−5,^ respectively and identified optimal values for these parameters. The feature selection was performed based on accuracy value in differentiating three response groups using a sequential forward selection (SFS) method which learns that which features are most informative at each time step, choosing the next features based on already selected features and the internal “belief” of the classifier. Prior to classification analysis, the estimated data set was randomly subsampled into 10 subsets, such that each subset had equal number of the CR, PR and NR population members, which was required to account for the imbalance that existed in the data (CR = 21, PR = 52, NR = 23). For each iteration (number of sample = 63), optimal *C* and γ parameters selected by grid-search, the optimal multivariate QUS model were obtained by using sequential forward selection and results were validated using leave-one-out cross validation. Based on classification rules, six features were selected for each subset [31]. Finally, the optimal features were selected from feature histogram of all selected features of 10 subsets.

### Statistical analysis

Statistical tests were used to compare response groups, in terms of mean QUS and texture-based parameters. To determine the type of statistical test to use to compare the groups, a Shapiro-Wilk normality test was performed on each feature data set to determine whether it follows a normal distribution. For two response group comparisons: CR *vs* PR, CR *vs* NR, and PR *vs* NR at each scan time point, an unpaired *t*-test was completed for the datasets that passed the normality test, otherwise, Mann-Whitney unpaired test was used. For comparison of three response groups at each scan time point and also within the time points, a one-way ANOVA was performed for the datasets that passed the normality test; otherwise, the Kruskal-Wallis test was used. Recurrence free survivals for all three response populations were created by the Kaplan-Meier method to clarify the time dependent cumulative survival rate, and the curves were compared using a log-rank test. A value of p < 0.05 was considered to determine statistical significance.

## Results

### Patient characteristics

This locally advanced breast tumor response monitoring study was approved by the Sunnybrook Research Institute research ethics board. The number of LABC patients enrolled in this study was 96. The average age of patients was 49 ± 10 years (range, 29–80 years). The average tumor size along the longest axis was 5.9 ± 2.8 cm (range: 1.6–14 cm). Among ninety six patients, 89% had invasive ductal carcinoma and 5% of patients had lobular carcinoma. Nine had grade I tumor, thirty nine had grade II tumors, and forty seven had grade III tumors. Patients had a variety of neoadjuvant treatment plans with 63% of patients receiving combined anthracycline and taxane-based chemotherapy. In our patient population, 33% of patients had HER2+ tumors, 28% had triple negative and 38% had ER and/or PR +\ with HER2- tumor type. All HER2+ patients were treated with trastuzumab during standard chemotherapy. Patients were classified into one of three groups based on ultimate clinical and pathological response to neo-adjuvant chemotherapy treatment. The number of CR, PR and NR patients were 21, 52, and 23, respectively. Clinical and pathological characteristics of CR, PR and NR patient groups are summarized in Table 1. Patient characteristics and their ultimate response according to clinical and pathological reports of all patients are presented in S1 and S2 Tables. The mean initial tumor sizes of CR, PR and NR patients were 4.9, 6.2, and 6.1 cm, respectively and mean tumor shrinkage in these patient groups after treatment was 100%, 54%, and 0.05%, respectively. In the complete responder group, the number of patients having negative ER and PR expression was higher (71%, and 76%, respectively) than in the partial responder (36%, and 42%, respectively) and non-responder (17%, and 34%, respectively) groups. In contrast, the number of patients having HER2 negative expression was lower in the complete responder group (47%) than in partial (69%) and non-responder (73%) groups.

**Table 1:** Clinical and pathologic characteristics of LABC patients receiving neo-adjuvant chemotherapy.

Characteristics	CR (N = 21)	PR (N = 52)	NR (N = 23)	All (N = 96)
**Age**				
Mean ± std (year)	49 ± 8	50 ± 10	47 ± 11	49 ± 10
Range (cm)	31–62	32–80	29–66	29–80
**Menopause**				
Postmenopausal (%)	38.1	30.8	30.4	32.3
Premenopausal (%)	47.6	57.7	65.2	57.3
Perimenopausal (%)	9.5	3.8	4.4	5.2
Unknown (%)	4.8	7.7	0	5.2
**Tumor size before NAC**				
Mean + std (cm)	4.9 ± 2.2	6.2 ± 3.0	6.1 ± 2.8	5.9 ± 2.8
Range (cm)	2.3–10.0	2.0–14.2	1.6–11.7	1.6–14.2
**Histological feature**				
IDC (%)	95.3	96.2	78.2	91.7
ILC (%)	0	3.8	13.0	5.2
IMC (%)	4.7	0	8.7	3.1
**Tumor stage**				
T2 (%)	42.8	38.5	34.8	38.5
T3 (%)	42.8	40.4	43.5	41.7
T4 (%)	4.8	3.8	4.3	4.2
T4d (%)	4.8	7.7	8.7	7.3
Unknown (%)	4.8	9.6	8.7	8.3
**Nodal status**				
N0 (%)	14.3	23.0	30.5	22.9
N1 (%)	71.3	50.0	47.8	54.2
N2 (%)	4.8	13.5	4.3	9.4
N3 (%)	4.8	0	8.7	3.1
Unknown (%)	4.8	13.5	8.7	10.4
**Tumor grade**				
I (%)	4.8	11.5	8.7	9.4
II (%)	19.0	38.5	60.9	39.6
III (%)	71.4	46.2	30.4	47.9
Unknown (%)	4.8	3.8	0	3.1
**Molecular features**				
ER—(%)	71.4	36.5	17.4	39.6
ER + (%)	28.6	63.5	82.6	60.4
PR—(%)	76.2	42.3	34.8	47.9
PR + (%)	23.8	57.7	65.2	52.1
HER2 - (%)	47.6	69.2	78.3	66.7
HER2 + (%)	52.4	30.8	21.7	33.3
Triple negative	42.9	23.1	17.4	28.2
ER or PR + \ HER2 -	4.8	42.3	60.9	38.5
**Treatment**				
AC-T (%)	61.9	67.3	52.2	62.5
FEC-D (%)	28.6	21.2	34.8	26.0
Trastuzumab (%)	52.4	30.8	21.7	33.3
Others (%)	9.5	11.5	13.0	11.5
**Tumor size after NAC**				
Mean ± std (cm)	0	2.6 ± 2.9	5.9 ± 4.2	2.9 ± 3.9
Range (cm)	0	0.1–17.0	1.2–19.0	0–19.0

Abbreviations: NAC, neoadjuvant chemotherapy; CR, complete responder; PR, partial responder; NR, non-responder; IDC, invasive ductal carcinoma; IMC, invasive mammary carcinoma; ILC, invasive lobular carcinoma; ER, estrogen receptor; PR, progesterone receptor; HER2, human epidermal growth factor receptor 2; ACT, adriamycin, cytoxan and paclitaxel; FECD, 5-fluourouracil, epirubicin, cyclophosphamide and docetaxel.

### Quantitative ultrasound parameter

**Fig 1:**
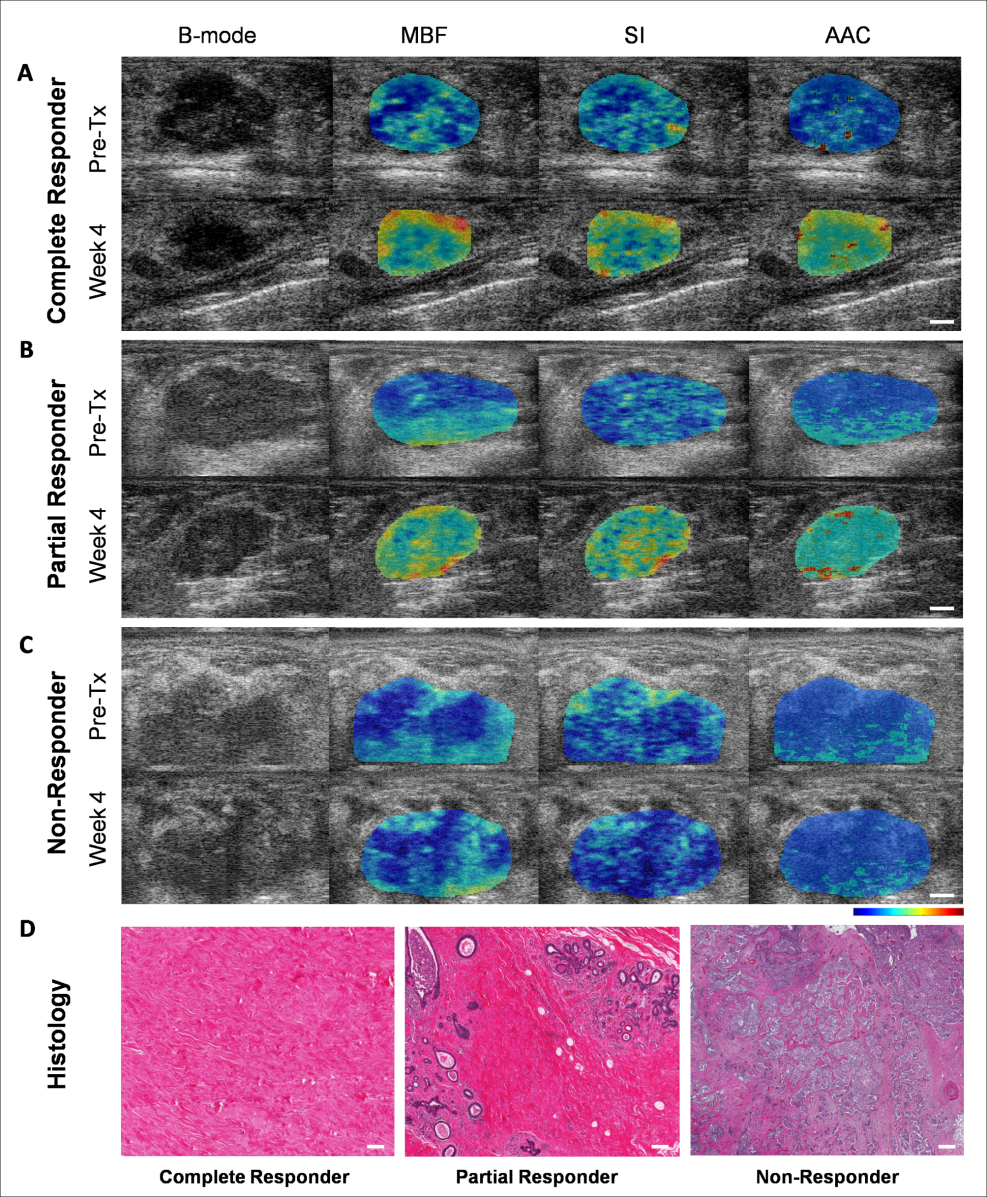
**B-mode and QUS parametric images MBF, SI, and AAC from a complete responder (A), partial responder (B), and non-responder (C) before start of NAC (Pre-Tx) and after 4 weeks of treatment. High magnification light microscope images of whole mount histopathology from complete, partial and non-responders (D). The scale bars in ultrasound images represent 5 mm. The color bars present scale for MBF parameter of -16 to 18 dBr, for SI parameter of -15 to 50 dBr, and for AAC parameter of 12 to 66 dB/cm**^**3**^**. The scale bar in histology represents 200 microns.** MBF, mid-band fit; SI, spectral intercept; AAC, average acoustic concentration, Pre-Tx, prior to treatment.

**Fig 2:**
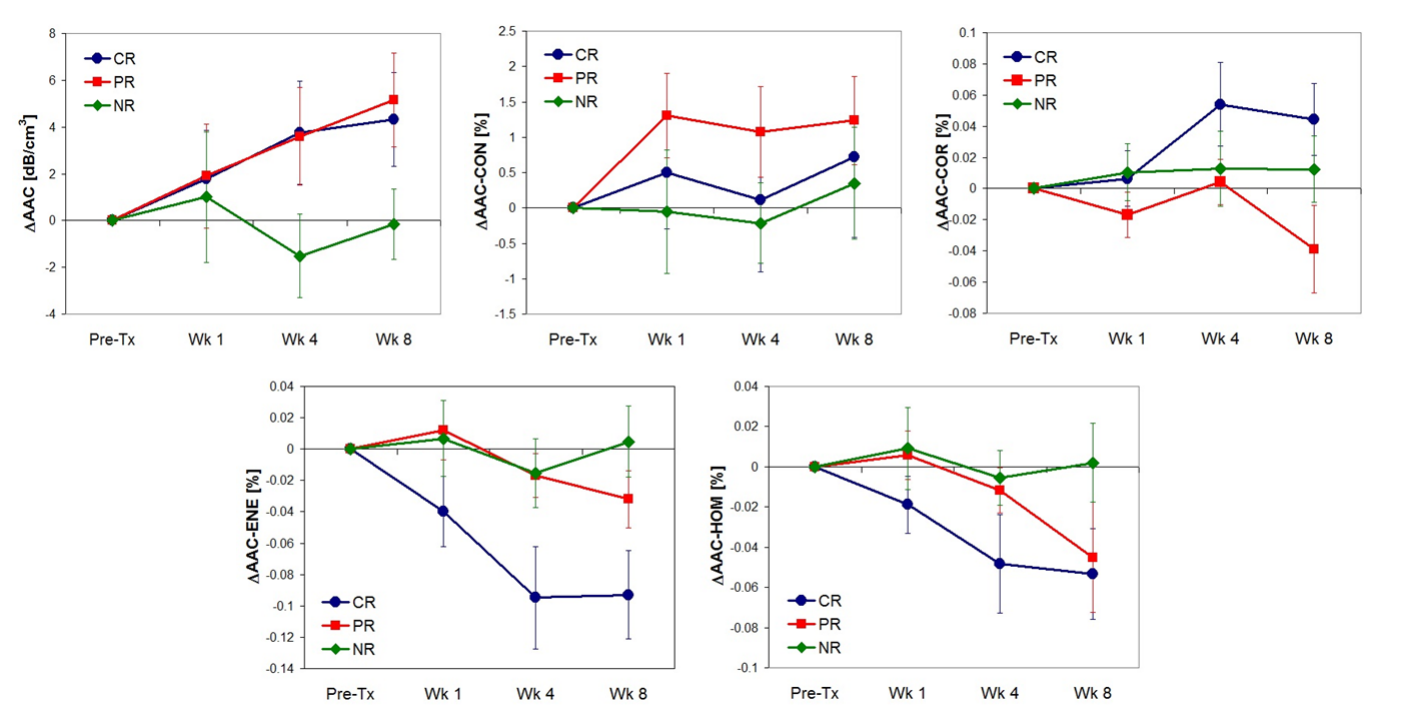
Average changes in average acoustic concentration and corresponding texture parameters measured in ultimate clinical and pathological complete, partial and non-responders at times before and after the start of treatment. AAC, average acoustic concentration; CON, contrast; COR, correlation; ENE, energy; HOM, homogeneity; CR, complete responder; PR, partial responder; NR, non-responder.

Parameters extracted from ultrasound radio frequency signals were MBF, SS, SI, ACE, SAS, ASD and AAC. Ultrasound B mode images, MBF, SI and AAC parametric images corresponding to complete (A), partial (B) and non responding (C) patients acquired prior to chemotherapy onset, and after four weeks of treatment, and high magnification H&E images (D) of corresponding histopathology for these three response types are displayed in Fig 1. The parameters MBF, SI, AAC were chosen to be presented here due to their dominant contribution in the three response group classification. Data demonstrate increases in the ultrasound backscatter power and changes in their textural patterns within the tumor region for complete and partial responding patients after the start of treatment by weeks 1–4. In contrast, parametric images exhibited no changes in their mean value and textural pattern in non-responding patients. Average changes in one of the quantitative ultrasound parameter, AAC and corresponding textural features of the CR, PR and NR group over the treatment period are presented in Fig 2. Histopathology analysis revealed no residual disease, and a complete degeneration of tumors cells in complete responding patients. In contrast, there was minimal residual disease in partial responders. Non-responding patients exhibited large deposits of residual disease. These changes in tumor macro- and microstructure were reflected in estimated ultrasound parameters.

### Multi-feature tumor response classification model

Statistical analysis using unpaired t-test to compare mean QUS values, texture-based parameters and molecular features estimated from two response groups: CR *vs* PR, CR *vs* NR and PR *vs* NR before treatment, and change in mean values of QUS parameters and textural features estimated during treatment are summarized in S3, S4, S5, and S6. Statistical analysis results revealed significant differences between CR and PR in SAS texture features such as SAS-CON0 (p = 0.008) and SAS-HOM0 (p = 0.015) (scatterer spacing contrast and homogeneity) and also differences between CR and NR (p = 0.007), and PR and NR in ACE0 (p = 0.047) (mean of attenuation) and MBF-ENE0 (p = 0.043) (energy of MBF) before treatment. Molecular features such as ER and HER2 which were determined from biopsy samples before treatment exhibited significant differences between CR and PR (p = 0.007) patient groups and also between CR and NR (p = 0.017) patient groups. During treatment at week 1, ΔSAS (p = 0.009) and ΔSAS-CON (p = 0.028) indicated differences between CR and PR. At weeks 4 and 8, changes in MBF (p = 0.037) and corresponding texture parameters such as ΔMBF-CON (p = 0.038) and ΔMBF-ENE (p = 0.033) were the dominant parameters in separating CR and PR from NR. Features exhibited significance differences between two response groups: CR *vs* PR, CR *vs* NR and PR *vs* NR before treatment, at week 1, 4 and 8 are presented in Table 2. Analysis using ANOVA with a Bonferroni correction demonstrated significant differences in changes of SI and texture parameters extracted from MBF, SI and AAC parametric maps acquired at week 1 and 8 in complete and partial responders. In contrast, none of the mean QUS and texture features from non-responders showed any changes after treatment initiation (S7 Table). Differences between responder, R (CR + PR) and NR were also investigated. Statistical analysis results revealed significant differences between R and NR mostly in their texture features such as MBF-ENE0 (p = 0.038), MBF-HOM0 (p = 0.032), and SI-CON0 (p = 0.048) (spectral intercept contrast) before treatment. During treatment at weeks 1 and 4, ΔACE (p = 0.032), ΔMBF-CON (p = 0.048), ΔMBF-ENE (p = 0.05), and ΔSS-ENE (p = 0.005) (spectral slope energy) indicated differences between these groups. At week 8, mean QUS values especially ultrasound backscatter intensity parameters, ΔMBF (p = 0.025), ΔSI (p = 0.048) and corresponding texture features, ΔMBF-ENE (p = 0.013), ΔMBF-HOM (p = 0.04), ΔSS-ENE (p = 0.048) revealed significant differences between R and NR.

**Table 2:** Features which exhibited significance differences between two response groups: CR *vs* PR, CR *vs* NR and PR *vs* NR.

Scan time	CR *vs* PR	CR *vs* NR	PR *vs* NR
Pre-Tx	SAS0, SAS-CON0, SAS-HOM0, HER 2	SAS-HOM0, HER2, ER	MBF-ENE0
Week 1	ΔSAS, ΔSAS-CON	ΔACE	ΔACE
Week 4	ΔAAC-ENE	ΔMBF-CON	ΔACE, ΔMBF-ENE
Week 8	Δ AAC-ENE	ΔMBF, ΔSI, ΔMBF-CON, ΔMBF-ENE, ΔMBF-HOM, ΔAAC-ENE,	ΔMBF, ΔMBF-ENE, ΔMBF-HOM, ΔSI-ENE,

In order to attempt to differentiate three response groups, a multi-feature SVM classification analysis was performed. Classification analysis performed on mean QUS + texture features, differentiated three response groups with accuracies of 54%, 60% and 59% at weeks 1, 4, and 8, respectively. Clinical receptors status alone differentiated the three response groups with accuracies of 38%, 37% and 50% at weeks 1, 4, and 8, respectively. However, the best classification accuracies were obtained with the combination of mean QUS values, texture and molecular features with accuracies of 79%, 86% and 83% at weeks 1, 4, and 8, respectively. All results were obtained after leave-one-out cross-validations. The optimal features were selected from feature histogram of selected features using a sequential feature selection method over 10 iterations. For example, the feature histogram for the week 8 data set is presented in Fig 3 and optimal features at this scan time were HER2 (8 occurrences), Δ SI (7 occurrences), Δ AAC-ENE (7 occurrences), ER (7 occurrences), Δ MBF (5 occurrences), and SAS-CON0 (4 occurrences). Optimal features selected for week 1, 4, 8 data sets are presented in Table 3 and features are arranged from higher to lower frequencies of occurrences over iterations.

**Fig 3:**
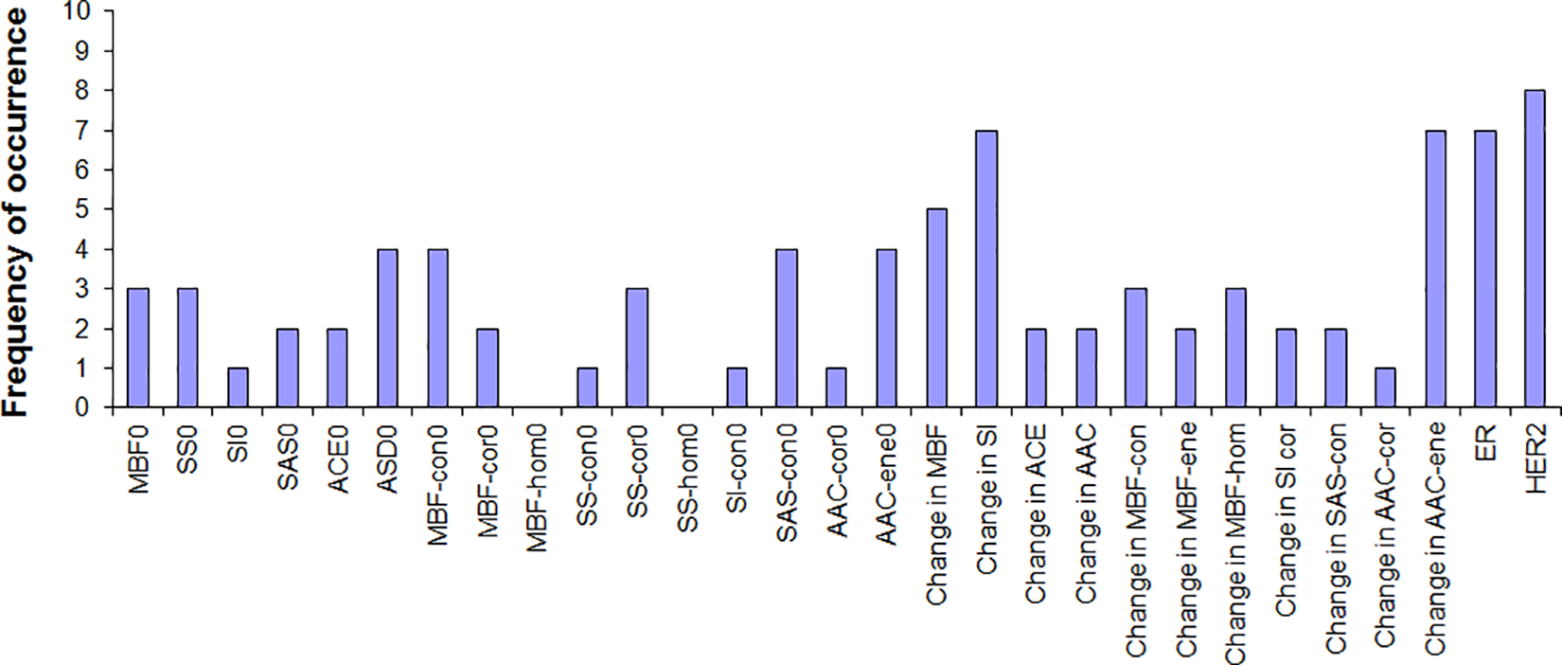
Feature histograms obtained from week 8 data set over 10 iterations of classifications. **In each iteration, best features were selected using sequential feature selection method and frequencies of occurrence of selected features over 10 iterations are presented.** MBF, mid-band fit; SS, spectral slope; SI, spectral intercept; SAS, spacing among scatterers; ASD, average scatterer diameter; ACE, attenuation co-efficient estimate; AAC, average acoustic concentration; ER, estrogen receptor; HER2, human epidermal growth factor receptor 2; CON, contrast; COR, correlation; ENE, energy; HOM, homogeneity; 0, parameter estimated prior to treatment.

**Table 3:** Selected features for three- type tumor response classification using sequential feature selection method. Features are arranged from higher to lower frequency of occurrence over 10 iterations.

Rank	Week 1	Week 4	Week 8
1	ER	MBF-CON0	HER2
2	AAC-COR0	Δ AAC-ENE	ER
3	SAS-CON0	HER2	Δ SI
4	Δ SAS-CON	MBF0	Δ MBF
5	Δ ACE	ASD0	Δ AAC-ENE
6	MBF-CON0	SS-COR0	SAS-CON0

#### Recurrence free survival

**Fig 4:**
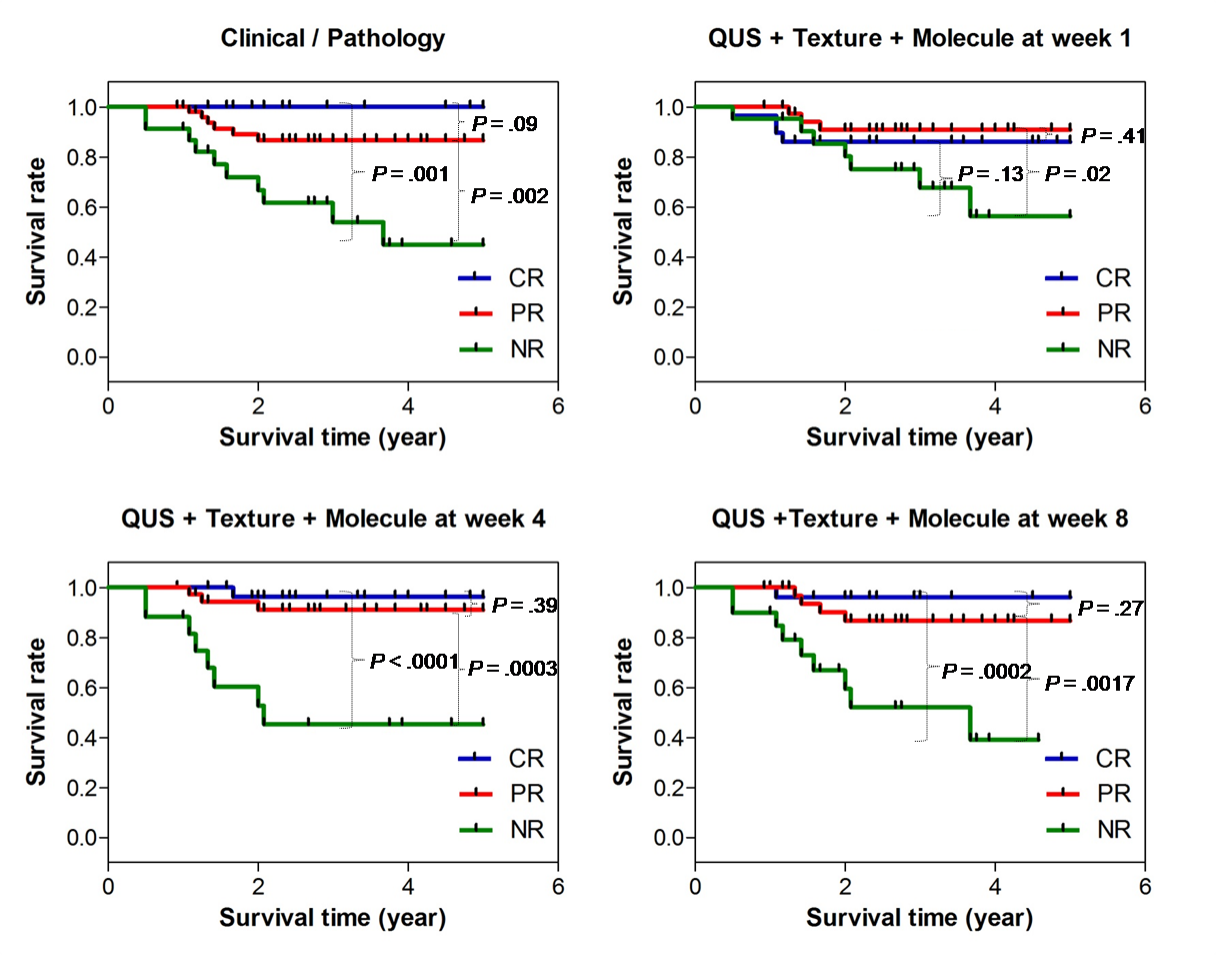
Recurrence free survival curves for chemotherapy treatment response based on ultimate clinical-pathological assessment, and comparison to predicted recurrence free survival curves when based on quantitative ultrasound, texture parameters and molecular features acquired at weeks 1, 4, and 8 after treatment. CR, complete responder; PR, partial responder; NR, non-responder; QUS + Texture + Molecule, combination of mean QUS values, texture and molecular features.

Recurrence free survival curves for chemotherapy treatment response based on clinical-pathological response and testing against combinations of mean QUS, texture, and molecular features at week 1, 4 and 8 after the start of treatment are presented in Fig 4. The 5-year recurrence free survival (RFS) rates for CR, PR, and NR patient groups were 100%, 89.7%, and 66.4%, respectively, with RFS of the pathological complete and partial responders significantly higher than that of the non-responders, as expected. However, no significant difference was found between the complete and partial responder groups enrolled in this study for the monitored follow up period. Whereas the classification model developed could differentiate all three response groups with an overall accuracy of 79% at week 1, the survival rate did not exhibit a significant difference between these groups at the follow-up period available. Similar to survival curves based on ultimate and pathological response, survival analysis based on estimated features at week 4 and 8 demonstrated a higher RFS for complete and partial responding patients compared to the non-responding patients.

## Discussion

In this study, we present the results of a clinical investigation on 96 locally advanced breast cancer patients receiving NAC, whose primary tumor responses were monitored using QUS and texture analysis techniques in conjunction with tumor molecular features early after the start of treatment. Seven quantitative ultrasound parameters and four texture features extracted from each QUS parametric maps were investigated from locally advanced breast tumors over the course of the treatment. The results exhibited considerably different trends for changes in mean QUS values and texture features for three different response groups (complete responders, partial responders, and patients with no response). Specifically, the majority of mean QUS parameters demonstrated similar trends for CR and PR after treatment initiation. Nevertheless, some of the associated QUS-based texture features exhibited different trends, but these differences were not significant. In contrast, QUS parameters and texture features determined from NR followed completely different trends from CR and PR during treatment.

Firstly, in order to understand the relationship between QUS-derived indicators of tumor microstructure and treatment response, the correlations of estimated QUS, texture and molecular features with treatment response were investigated. Statistical tests revealed ACE, and molecular features, ER and HER2 determined from CR and PR were significantly different from NR before treatment. ACE is related to tissue composition. This finding suggests the significance of tumor composition and molecular features as prognostic indicators of chemotherapy responsiveness. This result is consistent with a previous study that investigated relationship between baseline tumor oxygen (stO_2_) estimated using optical imaging techniques and pathological complete response in breast cancer patients treated with NAC [32]. That study reported that combining baseline stO_2_ and ER could differentiate pathological complete response and non-pathological complete response with a sensitivity of 100% and a specificity of 85%. In our study, texture characteristics of QUS parametric images especially, MBF and SI texture features revealed significant difference between R and NR before treatment. MBF and SI are strongly related to scatterer number density and their elastic properties. The range of scatter size, ASD (80–160 μm) estimated from tumor ultrasound data for the frequency bandwidth 4–9 MHz was comparable with lobule diameters observed from histopathology images. This suggests that tumors which responded to NAC (R) and tumors which did not respond to NAC (NR) have different lobule number density distributions. SAS and corresponding texture parameters estimated from CR and PR before start of treatment revealed significant differences. This suggests that spacing between adjacent lobules is different in complete and partial responding tumors. In addition to molecular features, these differences in CR, PR and NR microstructure arrangement respond differently to chemotherapy treatment.

Multi-feature discriminant analysis using a SVM classifier was performed on estimated features to improve the accuracy of a three type of response group classification. Sequential feature selection was used to select optimal features, and discriminant analysis was performed on selected features with cross validation using true labels identified by clinical/pathological examinations. The most accurate classifications were obtained by the combination of mean QUS values, texture and molecular features with accuracies of 79%, 86% and 83% at weeks 1, 4, and 8, respectively. Optimal features selected at these scan times are presented in Table 3. The optimal features selected to differentiate three response groups at week 1 were mostly texture parameters, especially pre-treatment texture features. More detectable changes in mean values of MBF, SI parametric maps were observed at week 8. These findings suggests that development of response in tumor cells is a gradual process which initially affects tissue micro-structure arrangement such as the spacing between lobules and later affects macro-structure such as lobule size, shape and their elastic properties and finally replaced with collagen and fibrotic deposition which reflect longer-term results of cell death process [33]. Ali et al [16] reported R and NR classification with the sensitivity 100% and specificity 100% early after NAC using combined mean QUS values and texture features. In that study, investigators used limited patient numbers and discriminant model was not validated. In our study, accuracies for R and NR classification using combined mean QUS values and texture features were 82%, 86%, and 90%, at week 1, 4, and 8 after NAC respectively. Adding molecular features with mean QUS values and texture parameters improved R and NR discrimination accuracies to 88%, 96%, and 95% at week 1, 4, and 8 after start of chemotherapy. The results obtained from binary and multiclass tumor responses classification in this study exhibit the importance of molecular features in determining the breast tumor response to NAC as described in previous studies [2,19].

Several other cancer response monitoring studies have been conducted using other functional imaging modalities. Specifically, several functional magnetic resonance imaging techniques such as dynamic contrast-enhanced (DCI)-MRI, diffusion weighted (DW)-MRI, blood oxygenation level-dependent (BOLD)-MRI, and MR elastography have been demonstrated in breast cancer characterization and their response to treatment early or after chemotherapy based on their ability in detecting changes in tumor microvasculature, cell density, hypoxia, metabolism, oxygenation and stiffness [34–38]. [^18^F]fluorodeoxyglucose positron emission tomography method has been demonstrated for detecting the pathological response of primary breast cancer after neoadjuvant chemotherapy treatment [22]. These modalities are often costly and require contrast agents to monitor tumor response to treatment. In contrast, the QUS techniques used in this study depend on internal contrast alterations scattered from differences in the acoustic impedance of tumor cells when they respond to treatment. Compared to previous QUS-based tumor response monitoring studies [14,15], the number of patients used in this study was relatively large and imbalances within tumor response group numbers were balanced by randomly subsampling the total population into several subsets before performing classification analysis and the discriminant model was cross validated to avoid over-fitting. Furthermore, we have shown that combining molecular feature with quantitative ultrasound technique significantly improved the discrimination between responder and non-responder groups and further helped to differentiate responder groups into complete and partial responders early after one week start of treatment.

Studies confirm that pathological response is a prognostic indicator for long term disease free and overall survivals [2,39]. In this study, the 5-year recurrence free survival (RFS) rates calculated for complete and partial responders were significantly higher than non-responder patient groups, as expected. However, no significant difference was found between the complete and partial responder groups in this study for the monitored follow up period. Miller et al [21] classified breast cancer patients into four groups such as complete response, strong partial response, weak partial response and tumor growth based on the ratio between residual in-breast disease divided by size on pre-NAC imaging and reported that complete response has significantly higher overall survival than strong and weak partial responses. It is possible that relatively small numbers of patients in CR compared to PR, and relatively short follow up times in this study limit the differences in RFS of CR and PR groups. Currently, primary tumor response to neoadjuvant chemotherapy is evaluated based on changes in tumor size, and histological examinations of the surgical specimens which take place typically months after chemotherapy treatment. Several previous studies have demonstrated that patients who do not respond to initial chemotherapy may benefit from salvage therapies: additional systemic chemotherapy or preoperative radiation or surgery [3–5]. The multi-feature tumor response classification model developed in this study by combination of quantitative ultrasound, texture and molecular features acquired early within one week after start of treatment could differentiate pathological response groups with reasonable accuracy Therefore, such an early insight into patient outcomes could facilitate the decision of switching to a more effective therapy for treatment-refractory patients or even shifting to a salvage therapy, before it is potentially too late. One of the limitations in our study is that all patient data investigated in this study were collected from a single institution and some patients did not have MRI imaging, thus forcing us to substitute ultrasound or CT imaging measurements which may not be precisely comparable although for response purposes are taken as clinically equivalent.

Several studies demonstrated significant difference in response and survival of different molecular subtype breast cancers: HER2+, triple negative, and ER and/or PR + with HER2- to chemotherapy treatment [2,20,40]. Therefore, it would be interesting to investigate the potential impact of molecular subtypes of breast cancer on therapy response evaluation using QUS and texture biomarkers obtained at different times after the start of treatment. 5 years disease free survival rate calculated for such three molecular subtypes breast cancer from our patient population did not exhibit significant difference. Accuracies for detecting molecular subtypes of breast cancer using QUS and texture biomarkers acquired before treatment, at weeks 1, 4, and 8 after treatment were 51.1%, 52.3%, 53.0%, and 53.7%, respectively. Therefore, larger cohort of patients with enough number of cases for each subtype will be required for such study to permit a more accurate cross-validated evaluation.

In conclusion, given the importance of detecting and evaluating the early tumor response to standard treatment many new techniques are being developed. We find quantitative ultrasound technique which has ability to detect tumor microstructure in cellular level to be particularly attractive given it simplicity and low cost and does not require the injection of any exogenous contrast agent. In terms of accuracy, it remains to be seen whether our preliminary observations are truly significant in a larger cohort of patients. The correlation between tumor response predicted based on quantitative ultrasound technique and histopathology analysis suggests that this may be an excellent, noninvasive, low cost tool to predict tumor response early after start of the treatment for oncologists. Validation of our multi-class tumor response classification approach, both through multi-site studies, larger patient population, and longer follow-up times would be useful to further support our findings.

## Supporting information

S1 Table All patient characteristics and pathological response results. (PDF)

S2 Table Ultimate responses of all patients according to the clinical and pathological reports. (PDF)

S3 Table Summary of p values obtained from statistical tests of significance carried out for mean QUS, texture, and molecular features estimated from two response groups before treatment using unpaired t-test. (PDF)

S4 Table Summary of p values obtained from statistical tests of significance carried out for change in mean QUS and texture features estimated from two response groups at week 1 after the treatment using unpaired t-test. (PDF)

S5 Table Summary of p values obtained from statistical tests of significance carried out for change in mean QUS and texture features estimated from two response groups at week 4 after the treatment using unpaired t-test. (PDF)

S6 Table Summary of p values obtained from statistical tests of significance carried out for change in mean QUS and texture features estimated from two response groups at week 8 after the treatment using unpaired t-test. (PDF)

S7 Table Summary of p values from statistical tests of significance carried out for changes in estimated parameters using ANOVA test over treatment times for all response groups and also over treatment responses. (PDF)

S8 Table Summary of p values obtained from statistical tests of significance carried out for change in mean QUS and texture features estimated from CR at two different scan time point using paired t-test. (PDF)

S9 Table Summary of p values obtained from statistical tests of significance carried out for change in mean QUS and texture features estimated from PR at two different scan time point using paired t-test. (PDF)

S10 Table Summary of p values obtained from statistical tests of significance carried out for change in mean QUS and texture features estimated from NR at two different scan time point using paired t-test. (PDF)
