# Supplementary material for: Response monitoring of breast cancer patients receiving neoadjuvant chemotherapy using quantitative ultrasound, texture, and molecular features
Source: PLoS One. 2018 Jan 3;13(1):e0189634. doi: 10.1371/journal.pone.0189634 (PMC5751990; doi:10.1371/journal.pone.0189634)
Supplement: S1 Table — (PDF) [file pone.0189634.s001.pdf]

S1 Table. Patient characteristics

No.	Age	Pre-treatment tumour size (cm)	Menopausal status	Histology	Grade	Tumour stage	Nodal status	ER/PR/HER2	Treatment
1	55	5.40	n/a	IDC	I	T4	N0	- - +	FECD + TRA
2	53	7.30	pre	IDC	III	T3	N2	+ + -	EDP
3	43	5.30	post	IDC	II	T3	N1	+ + +	TC + TRA
4	67	10.00	pre	IDC	III	T4d	N1	- - -	ACD
5	50	4.60	pre	IDC	III	T2	N1	+ + +	ACD + TRA
6	33	5.00	pre	ILC	III	T2	N0	+ + -	ACT
7	33	8.00	post	IDC	III	T3	N1	+++	ACT + TRA
8	48	4.90	pre	IDC	III	T4	N1	+ + -	ACD
9	36	5.80	pre	IDC	III	T3	N3	+ + -	ACD
10	40	4.40	post	IDC	III	T2	N0	- - -	ACT
11	62	10.00	post	IDC	II	T3	N1	- - -	D XRT
12	59	6.00	post	IDC	II	T3	N0	- - -	ACT
13	38	9.20	pre	IDC	III	T3	N1	+ + -	ACT
14	48	4.30	pre	IDC	III	T3	N2	+ + +	ACT + TRA
15	50	5.00	pre	IDC	III	T2	N2	- - -	ACT
16	49	12.00	peri	IDC	III	T3	N2	- - +	ACT + TRA
17	47	7.00	pre	IDC	III	T3	N2	- - -	ACT
18	40	3.00	post	IDC	II	T2	N1	- + +	ACT + TRA
19	56	3.20	pre	IDC	N/A	T2	N1	- + +	ACT + TRA
20	50	5.60	pre	IDC	III	T3	N1	- - +	ACT + TRA
21	52	4.10	pre	IDC	III	T2	N1	+ + -	ACT
22	44	9.90	pre	IDC	II	T3	N1	+ + +	ACT + TRA
23	38	9.00	post	IDC	III	T4	N2	+ + -	ACT
24	58	2.00	pre	IDC	II	T3	N0	- - -	ACT
25	N/A	5.90	pre	IDC	II	N/A	N/A	- - -	ACT
26	38	2.60	pre	IDC	II	T2	N0	- - +	ACT + TRA
27	47	9.90	post	IDC	III	T4d	N0	+ + -	ACT

28	57	5.50	post	IDC	I	T3	N1	- - -	ACT
29	47	7.40	pre	IDC	III	T3	N1	- - +	ACT + TRA
30	55	12.80	pre	IDC	I	T3	N1	- - -	ACT
31	33	7.00	pre	IDC	II	T3	N2	+ + +	ACT + TRA
32	38	2.50	pre	IDC	II	T2	N0	- - -	ACT
33	N/A	6.00	pre	IDC	III	T3	N1	+ + +	ACT + TRA
34	55	10.50	post	IDC	II	T3	N1	- - -	ACT
35	60	8.00	post	IDC	II	T3	N1	+ - +	FECD + TRA
36	55	N/A	n/a	IDC	III	T4d	?	+ + -	FECD
37	37	3.60	pre	IDC	III	T2	N1	+ + -	ACT
38	N/A	9.00	pre	IDC	II	T4d	N1	+ + +	ACT + TRA
39	N/A	3.60	peri	IDC	II	T2	N1	+ + -	TC
40	55	1.60	pre	IMC	II	T2	N1	+ - -	FECD
41	50	7.30	post	IDC	II	T3	N1	+ - -	FECD
42	55	3.40	n/a	IDC	II	T2	N/A	- - -	TC
43	44	3.50	pre	IDC	II	T2	N1	- - -	FECD
44	60	9.00	post	ILC	III	T3	N1	+ - -	FECD
45	64	8.70	post	ILC	I	T4d	N3	+ + -	FECD
46	52	2.60	post	IDC	I	T2	N0	- - -	FECD
47	47	14.20	post	IDC	I	N/A	N/A	- - -	FECD
48	56	7.00	pre	IDC	III	T3	N2	+ + +	ACT + TRA
49	45	2.30	post	IDC	III	T4d	N1	+ + +	FECD + TRA
50	59	4.90	post	IDC	III	T2	N0	+ + -	FECD
51	66	5.20	post	IDC	III	T3	N1	+ + +	TC + TRA
52	49	2.10	pre	IDC	III	T2	N0	+ + +	ACT + TRA
53	39	6.30	pre	IDC	II	T4d	N1	+ + -	FECD
54	31	6.30	pre	IDC	II	T3	N0	- - -	ACT
55	58	5.20	post	IDC	II	T3	N0	+ + +	ACT + TRA
56	51	4.00	pre	IMC	III	T2	N1	- - +	TC + TRA
57	45	4.00	pre	IDC	II	T2	N1	+ + -	ACT

58	29	4.20	pre	IDC	III	T2	N1	+ + -	ACT
59	80	3.90	post	IDC	II	T2	N1	+ - +	ACT + TRA
60	43	9.60	pre	IDC	II	T3	N1	++-	FECD
61	66	3.00	post	IDC	III	T2	N1	+ - +	FECD + TRA
62	39	5.00	pre	IDC	N/A	T2	N1	- - -	ACT
63	41	11.70	pre	IDC	II	T3	N2	+ + +	ACT + TRA
64	54	8.80	post	IDC	II	T4	N1	- - -	FECD
65	48	3.50	pre	IDC	III	T2	N0	+ + -	ACT
66	58	3.90	peri	IDC	III	T2	N0	+ - -	ACT
67	64	3.00	post	IDC	III	T2	N0	+ + -	FECD
68	48	2.40	peri	IDC	II	T2	N3	- - -	ACT
69	41	7.90	pre	IDC	III	T3	N1	+ + +	ACT + TRA
70	43	6.60	pre	IDC	II	T3	N0	+ + -	ACT
71	39	4.80	pre	IDC	II	T2	N1	- - -	AC
72	70	4.30	post	IDC	II	T2	N1	+ + -	FECD
73	52	4.20	post	IDC	II	T2	N1	- - +	ACT + TRA
74	54	5.60	n/a	IDC	I	T3	N1	- - +	ACT + TRA
75	51	2.20	pre	IDC	II	T2	N1	+ + -	ACT
76	56	3.10	post	IDC	II	T2	N1	+ - +	FECD + TRA
77	32	4.00	pre	IDC	III	T2	N1	+ + -	ACT
78	42	2.20	pre	ILC	III	T2	N1	+ + -	ACT
79	53	2.30	peri	IDC	II	T3	N0	+ + -	FECD
80	42	3.10	pre	IDC	II	T2	N1	- - -	ACT
81	31	3.50	pre	IDC	II	T3	N1	- - -	ACT
82	33	5.60	pre	IDC	I	T3	N1	- - -	ACT
83	46	8.10	pre	IDC	III	T3	N0	- - +	TC + TRA
84	53	5.30	pre	IDC	N/A	T3	N1	- - -	FECD
85	59	5.30	post	IDC	III	T3	N1	+ + +	FECD + TRA
86	38	10.90	pre	IDC	III	T3	N0	+ + -	FECD
87	73	3.30	post	IDC	III	T2	N0	+ - -	ACT

88	42	4.50	pre	IDC	II	T2	N1	- - -	ACT
89	48	5.60	pre	ILC	III	T3	N0	+ + -	FECD
90	N/A	7.70	post	IDC	II	T3	N1	+ - -	ACT
91	42	4.50	n/a	IDC	III	N/A	N/A	+ + -	ACT
92	N/A	9.00	pre	IDC	III	N/A	N/A	- - -	ACT
93	60	7.20	post	IDC	III	N/A	N/A	+ + +	ACT + TRA
94	42	6.30	pre	IDC	III	N/A	N/A	+ + -	FECD
95	47	10.40	pre	IDC	III	N/A	N/A	+ + -	ACT
96	56	11.70	pre	IMC	I	N/A	N/A	- - -	ACT

Abbreviations: IDC, invasive ductal carcinoma; invasive mammary carcinoma; ILC, invasive lobular carcinoma; ER, estrogen receptor; PR, progesterone receptor; HER2, human epidermal growth factor receptor 2; ACT, adriamycin, cytoxan and paclitaxel; FECD, 5-fluourouracil, epirubicin, cyclophosphamide and docetaxel; TRA, trastuzumab.
