# Supplementary material for: Response monitoring of breast cancer patients receiving neoadjuvant chemotherapy using quantitative ultrasound, texture, and molecular features
Source: PLoS One. 2018 Jan 3;13(1):e0189634. doi: 10.1371/journal.pone.0189634 (PMC5751990; doi:10.1371/journal.pone.0189634)
Supplement: S2 Table — (PDF) [file pone.0189634.s002.pdf]

S2 Table. Ultimate responses of all patients according to the clinical and pathological reports

No.	Post treatment tumour size (cm)	# Examined lymph nodes	# Involved lymph nodes	Miller-Payne	Clinical/ Pathology Response
1	0.00	17	0	5	CR
2	7.00	19	1	3	PR
3	2.70	R: 22 L:4	R:5 L:0	2	NR
4	1.60	7	7	3	PR
5	0.00	29	0	5	CR
6	1.40	11	2	3	PR
7	0.00	20	0	5	CR
8	1.40	18	7	3	PR
9	11.40	N/A	N/A	1	NR
10	2.00	12	0	3	PR
11	0.00	17	0	5	CR
12	2.60	10	1	3	PR
13	4.50	24	7	2	NR
14	5.00	N/A	N/A	2	NR
15	4.00	10	0	3	PR
16	2.00	11	1	3	PR
17	0.00	10	1	5	CR
18	0.00	21	0	5	CR
19	0.20	19	0	4	PR
20	0.10	14	0	4	PR
21	0.00	2	0	5	PR
22	2.00	21	3	4	PR
23	2.00	11	2	3	PR
24	0.20	11	0	4	PR
25	0.00	2	0	5	CR
26	0.00	N/A	N/A	5	CR

27	18.00	14	0	2	NR
28	0.00	R:18 L:12	R:6 L:4	5	CR
29	0.00	20	2	5	CR
30	17.00	20	1	3	PR
31	7.40	22	21	3	PR
32	3.80	12	1	1	NR
33	4.80	31	3	2	NR
34	0.10	4	2	4	PR
35	0.00	10	1	5	CR
36	N/A	28	0	3	PR
37	2.20	N/A	N/A	2	NR
38	1.20	31	2	3	PR
39	1.70	10	6	3	PR
40	1.20	1	1	2	NR
41	2.10	25	1	3	PR
42	1.80	11	0	3	PR
43	0.00	4	0	5	PR
44	8.00	20	0	2	NR
45	19.00	14	11	1	NR
46	2.40	7	5	3	PR
47	6.70	4	0	3	PR
48	8.40	34	2	3	PR
49	0.00	16	4	5	CR
50	2.80	17	2	3	PR
51	4.00	11	4	2	NR
52	1.60	27	7	3	PR
53	1.70	19	0	3	PR
54	12.60	25	2	1	NR
55	3.40	21	6	3	PR
56	0.00	18	0	5	CR

57	3.00	28	0	2	NR
58	4.00	12	4	2	NR
59	<1	21	14	3	PR
60	3.00	6	2	4	PR
61	2.40	22	0	2	NR
62	5.00	16	1	3	PR
63	1.30	19	4	3	PR
64	2.50	23	1	2	NR
65	4.00	7	0	2	NR
66	3.30	20	5	3	NR
67	0.50	7	2	3	PR
68	0.00	13	0	5	CR
69	0.20	11	0	4	PR
70	4.00	14	0	3	PR
71	0.00	13	1	5	CR
72	2.00	2	0	3	PR
73	0.00	32	0	5	CR
74	0.20	19	0	4	PR
75	0.50	17	0	3	PR
76	0.00	14	1	5	CR
77	1.70	29	0	3	PR
78	1.10	21	0	4	PR
79	0.00	16	5	5	CR
80	0.00	2	0	5	CR
81	0.00	19	0	5	CR
82	2.70	37	0	3	PR
83	1.60	37	14	4	PR
84	0.00	1	0	5	CR
85	1.50	14	0	4	PR
86	4.90	4	0	2	NR

87	0.20	26	11	4	PR
88	2.00	20	0	3	PR
89	11.00	4	0	2	NR
90	0.00	6	1	4	PR
91	3.50	2	0	3	PR
92	3.90	12	8	3	PR
93	1.00	7	2	4	PR
94	8.40	12	11	2	NR
95	4.50	20	9	4	PR
96	9.40	23	16	2	NR

Abbreviations: CR, complete responder; PR, partial responder; NR, non-responder.
