# Supplementary material for: Response monitoring of breast cancer patients receiving neoadjuvant chemotherapy using quantitative ultrasound, texture, and molecular features
Source: PLoS One. 2018 Jan 3;13(1):e0189634. doi: 10.1371/journal.pone.0189634 (PMC5751990; doi:10.1371/journal.pone.0189634)
Supplement: S3 Table — (PDF) [file pone.0189634.s003.pdf]

S3 Table. Summary of p values obtained from statistical tests of significance carried out for mean QUS, texture, and molecular features estimated from two response groups before treatment using unpaired t-test.

Features	CR vs PR	CR vs NR	PR vs NR
ER	0.698	0.012*	0.771
PR	0.329	0.095	0.550
HER2	0.007*	0.017*	0.876
MBF0(dBr)	0.350	0.83	0.380
SS0(dB/MHz)	0.232	0.33	0.130
SI0(dBr)	0.520	0.60	0.940
SAS0(mm)	0.022*	0.093	0.989
ACE0(dB/cm-MHz)	0.413	0.426	0.047*
ASD0(um)	0.023*	0.320	0.132
AAC0(dB/cm^3)	0.088	0.285	0.389
MBF con0	0.536	0.364	0.589
MBF cor0	0.545	0.423	0.659
MBF ene0	0.686	0.057	0.043*
MBF hom0	0.852	0.179	0.072
SS con0	0.777	0.174	0.169
SS cor0	0.548	0.631	0.251
SS ene0	0.981	0.493	0.425
SS hom0	0.786	0.679	0.441
SI con0	0.469	0.080	0.077
SI cor0	0.875	0.179	0.097
SI ene0	0.336	0.135	0.300
SI hom0	0.667	0.264	0.304
SAS con0	0.008	0.060	0.917
SAS cor0	0.603	0.420	0.667
SAS ene0	0.073	0.087	0.273
SAS hom0	0.015*	0.044*	0.734
ASD con0	0.323	0.910	0.302
ASD cor0	0.273	0.840	0.207
ASD ene0	0.198	0.287	0.791
ASD hom0	0.908	0.479	0.289
AAC con0	0.217	0.572	0.394
AAC cor0	0.057	0.315	0.203
AAC ene0	0.077	0.059	0.818
AAC hom0	0.247	0.179	0.691

* Statistically significant ($p < 0.05$).
