# Supplementary material for: Response monitoring of breast cancer patients receiving neoadjuvant chemotherapy using quantitative ultrasound, texture, and molecular features
Source: PLoS One. 2018 Jan 3;13(1):e0189634. doi: 10.1371/journal.pone.0189634 (PMC5751990; doi:10.1371/journal.pone.0189634)
Supplement: S4 Table — (PDF) [file pone.0189634.s004.pdf]

S4 Table. Summary of p values obtained from statistical tests of significance carried out for change in mean QUS and texture features estimated from two response groups at week 1 after the treatment using unpaired t-test.

Features	CR vs PR	CR vs NR	PR vs NR
Δ MBF(dBr)	0.978	0.645	0.565
Δ SS(dB/MHz)	0.290	0.930	0.221
Δ SI(dBr)	0.539	0.685	0.833
Δ SAS(mm)	0.009*	0.569	0.581
Δ ACE(dB/cm-MHz)	0.623	0.092	0.007*
Δ ASD(um)	0.254	0.937	0.193
Δ AAC(dB/cm^3)	0.361	0.781	0.526
Δ MBF con	0.763	0.225	0.087
Δ MBF cor	0.618	0.423	0.145
Δ MBF ene	0.427	0.319	0.707
Δ MBF hom	0.661	0.499	0.162
Δ SS con	0.440	0.176	0.376
Δ SS cor	0.836	0.562	0.659
Δ SS ene	0.525	0.087	0.134
Δ SS hom	0.687	0.245	0.289
Δ SI con	0.760	0.274	0.277
Δ SI cor	0.733	0.276	0.104
Δ SI ene	0.345	0.096	0.171
Δ SI hom	0.806	0.245	0.188
Δ SAS con	0.028*	0.219	0.549
Δ SAS cor	0.379	0.250	0.584
Δ SAS ene	0.333	0.832	0.531
Δ SAS hom	0.020	0.457	0.680
Δ ASD con	0.618	0.286	0.392
Δ ASD cor	0.769	0.687	0.449
Δ ASD ene	0.064	0.182	0.648
Δ ASD hom	0.131	0.061	0.630
Δ AAC con	0.811	0.341	0.335
Δ AAC cor	0.503	0.573	0.170
Δ AAC ene	0.119	0.472	0.354
Δ AAC hom	0.240	0.672	0.525

* Statistically significant ($p < 0.05$).
