# Supplementary material for: Response monitoring of breast cancer patients receiving neoadjuvant chemotherapy using quantitative ultrasound, texture, and molecular features
Source: PLoS One. 2018 Jan 3;13(1):e0189634. doi: 10.1371/journal.pone.0189634 (PMC5751990; doi:10.1371/journal.pone.0189634)
Supplement: S5 Table — (PDF) [file pone.0189634.s005.pdf]

S5 Table. Summary of p values obtained from statistical tests of significance carried out for change in mean QUS and texture features estimated from two response groups at week 4 after the treatment using unpaired t-test.

Features	CR vs PR	CR vs NR	PR vs NR
Δ MBF(dBr)	0.845	0.168	0.089
Δ SS(dB/MHz)	0.294	0.842	0.156
Δ SI(dBr)	0.540	0.096	0.428
Δ SAS(mm)	0.431	0.636	0.209
Δ ACE(dB/cm-MHz)	0.758	0.284	0.031*
Δ ASD(um)	0.303	0.809	0.117
Δ AAC(dB/cm^3)	0.668	0.569	0.072
Δ MBF con	0.535	0.051*	0.644
Δ MBF cor	0.576	0.551	0.561
Δ MBF ene	0.936	0.161	0.042*
Δ MBF hom	0.968	0.185	0.100
Δ SS con	0.319	0.185	0.674
Δ SS cor	0.774	0.772	0.982
Δ SS ene	0.587	0.222	0.429
Δ SS hom	0.674	0.407	0.708
Δ SI con	0.596	0.737	0.837
Δ SI cor	0.657	0.967	0.615
Δ SI ene	0.946	0.578	0.562
Δ SI hom	0.957	0.903	0.859
Δ SAS con	0.691	0.667	0.987
Δ SAS cor	0.434	0.627	0.214
Δ SAS ene	0.902	0.441	0.717
Δ SAS hom	0.515	0.251	0.856
Δ ASD con	0.927	0.396	0.383
Δ ASD cor	0.805	0.706	0.884
Δ ASD ene	0.829	0.773	0.464
Δ ASD hom	0.952	0.805	0.721
Δ AAC con	0.509	0.659	0.214
Δ AAC cor	0.162	0.334	0.795
Δ AAC ene	0.033*	0.079	0.851
Δ AAC hom	0.226	0.183	0.706

* Statistically significant ($p < 0.05$).
