# Supplementary material for: Response monitoring of breast cancer patients receiving neoadjuvant chemotherapy using quantitative ultrasound, texture, and molecular features
Source: PLoS One. 2018 Jan 3;13(1):e0189634. doi: 10.1371/journal.pone.0189634 (PMC5751990; doi:10.1371/journal.pone.0189634)
Supplement: S6 Table — (PDF) [file pone.0189634.s006.pdf]

S6 Table. Summary of p values obtained from statistical tests of significance carried out for change in mean QUS and texture features estimated from two response groups at week 8 after the treatment using unpaired t-test.

Features	CR vs PR	CR vs NR	PR vs NR
∆ MBF(dBr)	0.738	0.041*	0.034*
∆ SS(dB/MHz)	0.260	0.791	0.297
∆ SI(dBr)	0.222	0.026*	0.185
∆ SAS(mm)	0.465	0.075	0.924
∆ ACE(dB/cm-MHz)	0.717	0.768	0.409
∆ ASD(um)	0.168	0.863	0.145
∆ AAC(dB/cm^3)	0.872	0.514	0.161
∆ MBF con	0.530	0.008*	0.600
∆ MBF cor	0.548	0.389	0.507
∆ MBF ene	0.995	0.042*	0.016*
∆ MBF hom	0.415	0.034*	0.056
∆ SS con	0.709	0.736	0.456
∆ SS cor	0.204	0.287	0.358
∆ SS ene	0.023*	0.978	0.182
∆ SS hom	0.170	0.575	0.204
∆ SI con	0.904	0.817	0.710
∆ SI cor	0.218	0.313	0.351
∆ SI ene	0.245	0.502	0.048*
∆ SI hom	0.276	0.934	0.245
∆ SAS con	0.608	0.677	0.883
∆ SAS cor	0.063	0.178	0.409
∆ SAS ene	0.712	0.428	0.966
∆ SAS hom	0.358	0.886	0.329
∆ ASD con	0.622	0.764	0.380
∆ ASD cor	0.181	0.248	0.350
∆ ASD ene	0.565	0.801	0.605
∆ ASD hom	0.428	0.477	0.214
∆ AAC con	0.900	0.548	0.342
∆ AAC cor	0.095	0.297	0.271
∆ AAC ene	0.043*	0.007*	0.236
∆ AAC hom	0.958	0.051	0.201

* Statistically significant ($p < 0.05$).
