# Supplementary material for: Response monitoring of breast cancer patients receiving neoadjuvant chemotherapy using quantitative ultrasound, texture, and molecular features
Source: PLoS One. 2018 Jan 3;13(1):e0189634. doi: 10.1371/journal.pone.0189634 (PMC5751990; doi:10.1371/journal.pone.0189634)
Supplement: S7 Table — (PDF) [file pone.0189634.s007.pdf]

**S7 Table. Summary of p values from statistical tests of significance carried out for changes in estimated parameters using ANOVA test over treatment times for all response groups and also over treatment responses.**

| Features | Over treatment time | | | Over responses | | |
|---|---|---|---|---|---|---|
| | **CR** | **PR** | **NR** | **Week 1** | **Week 4** | **Week 8** |
| MBF(dBr) | 0.499 | 0.132 | 0.269 | 0.908 | 0.328 | 0.029* |
| SS(dB/MHz) | 0.465 | 0.642 | 0.988 | 0.340 | 0.586 | 0.407 |
| SI(dBr) | 0.050* | 0.414 | 0.582 | 0.825 | 0.281 | 0.042* |
| SAS(mm) | 0.458 | 0.714 | 0.991 | 0.388 | 0.199 | 0.406 |
| ACE(dB/cm-MHz) | 0.807 | 0.144 | 0.856 | 0.041* | 0.339 | 0.585 |
| ASD(um) | 0.463 | 0.452 | 0.954 | 0.260 | 0.535 | 0.189 |
| AAC(dB/$cm^3$) | 0.980 | 0.193 | 0.398 | 0.514 | 0.713 | 0.484 |
| MBF con | 0.154 | 0.734 | 0.497 | 0.089 | 0.736 | 0.691 |
| MBF cor | 0.475 | 0.690 | 0.509 | 0.757 | 0.680 | 0.670 |
| MBF ene | 0.050* | 0.003* | 0.918 | 0.318 | 0.195 | 0.020* |
| MBF hom | 0.382 | 0.009 | 0.910 | 0.138 | 0.533 | 0.090 |
| SS con | 0.541 | 0.887 | 0.836 | 0.196 | 0.478 | 0.701 |
| SS cor | 0.524 | 0.055 | 0.778 | 0.479 | 0.827 | 0.267 |
| SS ene | 0.180 | 0.010* | 0.567 | 0.126 | 0.250 | 0.009* |
| SS hom | 0.569 | 0.025* | 0.767 | 0.183 | 0.696 | 0.170 |
| SI con | 0.870 | 0.953 | 0.952 | 0.524 | 0.623 | 0.866 |
| SI cor | 0.674 | 0.059 | 0.694 | 0.289 | 0.759 | 0.276 |
| SI ene | 0.432 | 0.023* | 0.941 | 0.188 | 0.376 | 0.086 |
| SI hom | 0.936 | 0.038* | 0.886 | 0.471 | 0.801 | 0.279 |
| SAS con | 0.942 | 0.438 | 0.674 | 0.158 | 0.496 | 0.970 |
| SAS cor | 0.631 | 0.032* | 0.862 | 0.304 | 0.520 | 0.165 |
| SAS ene | 0.927 | 0.898 | 0.744 | 0.424 | 0.670 | 0.836 |
| SAS hom | 0.872 | 0.153 | 0.746 | 0.374 | 0.46 | 0.382 |
| ASD con | 0.442 | 0.689 | 0.982 | 0.391 | 0.972 | 0.571 |
| ASD cor | 0.421 | 0.075 | 0.793 | 0.417 | 0.927 | 0.229 |
| ASD ene | 0.761 | 0.407 | 0.580 | 0.115 | 0.987 | 0.77 |
| ASD hom | 0.575 | 0.060 | 0.826 | 0.097 | 0.915 | 0.311 |
| AAC con | 0.667 | 0.521 | 0.992 | 0.403 | 0.740 | 0.677 |
| AAC cor | 0.050* | 0.338 | 0.955 | 0.443 | 0.199 | 0.119 |
| AAC ene | 0.015* | 0.224 | 0.839 | 0.287 | 0.024* | 0.029* |
| AAC hom | 0.184 | 0.047* | 0.988 | 0.497 | 0.241 | 0.348 |

* Statistically significant ($p < 0.05$).
