# Supplementary material for: Response monitoring of breast cancer patients receiving neoadjuvant chemotherapy using quantitative ultrasound, texture, and molecular features
Source: PLoS One. 2018 Jan 3;13(1):e0189634. doi: 10.1371/journal.pone.0189634 (PMC5751990; doi:10.1371/journal.pone.0189634)
Supplement: S8 Table — (PDF) [file pone.0189634.s008.pdf]

S8 Table. Summary of p values obtained from statistical tests of significance carried out for change in mean QUS and texture features estimated from CR at two different scan time point using paired t-test

Features	Wk1 vs Wk4	Wk1 vs Wk8	Wk4 vs Wk8
Δ MBF(dBr)	0.674	0.265	0.504
Δ SS(dB/MHz)	0.501	0.523	0.983
Δ SI(dBr)	0.272	0.121	0.415
Δ SAS(mm)	0.737	0.221	0.179
Δ ACE(dB/cm-MHz)	0.907	0.977	0.904
Δ ASD(um)	0.595	0.542	0.962
Δ AAC(dB/cm^3)	0.831	0.738	0.956
Δ MBF con	0.166	0.088	0.650
Δ MBF cor	0.198	0.051*	0.299
Δ MBF ene	0.571	0.260	0.647
Δ MBF hom	0.301	0.113	0.552
Δ SS con	0.461	0.788	0.374
Δ SS cor	0.871	0.418	0.583
Δ SS ene	0.132	0.799	0.076
Δ SS hom	0.436	0.705	0.294
Δ SI con	0.719	0.832	0.926
Δ SI cor	0.976	0.730	0.739
Δ SI ene	0.584	0.879	0.730
Δ SI hom	0.700	0.775	0.960
Δ SAS con	0.368	0.420	0.811
Δ SAS cor	0.363	0.600	0.781
Δ SAS ene	0.582	0.946	0.637
Δ SAS hom	0.739	0.511	0.747
Δ ASD con	0.586	0.450	0.840
Δ ASD cor	0.979	0.412	0.543
Δ ASD ene	0.522	0.656	0.776
Δ ASD hom	0.587	0.631	0.871
Δ AAC con	0.430	0.761	0.693
Δ AAC cor	0.149	0.206	0.781
Δ AAC ene	0.173	0.149	0.966
Δ AAC hom	0.306	0.165	0.873

* Statistically significant ($p < 0.05$).
