# Supplementary material for: Response monitoring of breast cancer patients receiving neoadjuvant chemotherapy using quantitative ultrasound, texture, and molecular features
Source: PLoS One. 2018 Jan 3;13(1):e0189634. doi: 10.1371/journal.pone.0189634 (PMC5751990; doi:10.1371/journal.pone.0189634)
Supplement: S9 Table — (PDF) [file pone.0189634.s009.pdf]

S9 Table. Summary of p values obtained from statistical tests of significance carried out for change in mean QUS and texture features estimated from PR at different scan time point using paired t-test

Features	Wk1 vs Wk4	Wk1 vs Wk8	Wk4 vs Wk8
Δ MBF(dBr)	0.608	0.468	0.841
Δ SS(dB/MHz)	0.340	0.752	0.649
Δ SI(dBr)	0.254	0.340	0.882
Δ SAS(mm)	0.044*	0.967	0.334
Δ ACE(dB/cm-MHz)	0.722	0.957	0.810
Δ ASD(um)	0.364	0.949	0.443
Δ AAC(dB/cm^3)	0.937	0.585	0.672
Δ MBF con	0.358	0.347	0.768
Δ MBF cor	0.304	0.280	0.699
Δ MBF ene	0.041*	0.003*	0.200
Δ MBF hom	0.167	0.018*	0.057*
Δ SS con	0.841	0.468	0.422
Δ SS cor	0.606	0.100	0.060
Δ SS ene	0.482	0.004*	0.034
Δ SS hom	0.953	0.046	0.050*
Δ SI con	0.681	0.716	0.482
Δ SI cor	0.315	0.124	0.050*
Δ SI ene	0.388	0.011*	0.083
Δ SI hom	0.998	0.061	0.059
Δ SAS con	0.614	0.542	0.840
Δ SAS cor	0.626	0.046	0.127
Δ SAS ene	0.518	0.544	0.844
Δ SAS hom	0.614	0.167	0.126
Δ ASD con	0.531	0.896	0.502
Δ ASD cor	0.449	0.140	0.065
Δ ASD ene	0.517	0.458	0.868
Δ ASD hom	0.861	0.067	0.071
Δ AAC con	0.787	0.933	0.854
Δ AAC cor	0.310	0.481	0.162
Δ AAC ene	0.218	0.098	0.504
Δ AAC hom	0.293	0.048*	0.147

* Statistically significant ($p < 0.05$).
