# Supplementary material for: Response monitoring of breast cancer patients receiving neoadjuvant chemotherapy using quantitative ultrasound, texture, and molecular features
Source: PLoS One. 2018 Jan 3;13(1):e0189634. doi: 10.1371/journal.pone.0189634 (PMC5751990; doi:10.1371/journal.pone.0189634)
Supplement: S10 Table — (PDF) [file pone.0189634.s010.pdf]

S10 Table. Summary of p values obtained from statistical tests of significance carried out for change in mean QUS and texture features estimated from NR at different scan time point using paired t-test

Features	Wk1 vs Wk4	Wk1 vs Wk8	Wk4 vs Wk8
Δ MBF(dBr)	0.430	0.135	0.366
Δ SS(dB/MHz)	0.939	0.748	0.788
Δ SI(dBr)	0.458	0.208	0.528
Δ SAS(mm)	0.413	0.526	0.693
Δ ACE(dB/cm-MHz)	0.855	0.409	0.560
Δ ASD(um)	0.989	0.705	0.693
Δ AAC(dB/cm^3)	0.643	0.278	0.532
Δ MBF con	0.933	0.739	0.770
Δ MBF cor	0.386	0.252	0.212
Δ MBF ene	0.887	0.729	0.674
Δ MBF hom	0.185	0.702	0.426
Δ SS con	0.362	0.376	0.875
Δ SS cor	0.249	0.197	0.904
Δ SS ene	0.219	0.501	0.633
Δ SS hom	0.124	0.262	0.775
Δ SI con	0.340	0.530	0.690
Δ SI cor	0.231	0.211	0.990
Δ SI ene	0.408	0.771	0.628
Δ SI hom	0.196	0.442	0.661
Δ SAS con	0.405	0.812	0.524
Δ SAS cor	0.377	0.766	0.276
Δ SAS ene	0.296	0.416	0.670
Δ SAS hom	0.425	0.752	0.529
Δ ASD con	0.513	0.809	0.633
Δ ASD cor	0.362	0.251	0.841
Δ ASD ene	0.717	0.506	0.707
Δ ASD hom	0.143	0.340	0.702
Δ AAC con	0.477	0.771	0.604
Δ AAC cor	0.879	0.949	0.835
Δ AAC ene	0.439	0.980	0.374
Δ AAC hom	0.431	0.774	0.573

* Statistically significant ($p < 0.05$).
